# Access to and Satisfaction with Basic Services in Informal Settlements: Results from a Baseline Assessment Survey

**DOI:** 10.3390/ijerph17124400

**Published:** 2020-06-19

**Authors:** Chipo Mutyambizi, Tholang Mokhele, Catherine Ndinda, Charles Hongoro

**Affiliations:** 1 Developmental, Capable and Ethical State, Human Sciences Research Council, Pretoria 0001, South Africa; chongoro@hsrc.ac.za; 2 eResearch Knowledge Centre, Human Sciences Research Council, Pretoria 0001, South Africa; tamokhele@hsrc.ac.za; 3 Human and Social Capabilities, Human Sciences Research Council, Pretoria 0001, South Africa; cndinda@hsrc.ac.za; 4 Department of Environmental Health, Tshwane University of Technology, Pretoria 0183, South Africa

**Keywords:** informal settlements, basic services, satisfaction, determinants, South Africa

## Abstract

Subjective responses of satisfaction with basic services delivery is an indicator of service delivery performance. This study provides an overview of the status of basic service delivery and determines the factors associated with service delivery satisfaction within informal settlements targeted for upgrading in South Africa. A multinomial logistic regression was used to analyze the relationship between satisfaction with basic services of water, sanitation, refuse and electricity with several predictors including individual factors, household factors, community factors and service-related factors. The most common source of drinking water, toilet facility and refuse disposal method were communal tap (55%) pit latrine (53%) and local authorities (34%), respectively. Approximately 52% of the respondents in the study reported not having access to electricity. Results also show that satisfaction in basic services delivery varies and is influenced by service-related factors. Interventions targeted at improving the quality of basic service provided are essential to meet the targets set out in the sustainable development goals.

## 1. Introduction

Access to basic services (water, sanitation, and energy) for the poor, who live in informal settlements, is both a local and global concern articulated in the sustainable development goals (SDGs). Out of the 17 SDGs, two specifically focus on access to and responsible use of basic services. SDG 6 seeks to ensure universal and equitable access to water and sanitation, SDG 7 addresses access to affordable, reliable, sustainable and modern energy for all [1]. The South African Government also recognizes that access to basic services is an important means of improving the lives of its people and local economic development. Basic services are defined as services that ensure a decent, acceptable standard of living in communities and facilitate the establishment of environments in which public health and safety can be promoted [2].

The provision of basic services has been a focus of the government since the transition to democracy in 1994. By 2010, about 90% of the population had access to water, 69% had access to sanitation, 64% had access to refuse removal, and 81% had access to electricity [3]. The Department of Human Settlements, which is responsible for the upgrading of informal settlements, in 2010, committed to support the government to ensure that 100% of the population had access to clean water, 92% accessed adequate sanitation, 75% accessed refuse removal, and 92% of the population was connected to the electricity grid [3]. The national levels of access to basic services conceals the inequalities that exist in between housing typologies (formal housing, traditional dwellings and informal settlements). The apartheid spatial planning that segregated the population in terms of race has also meant that access to basic services remains racialized [4]. Thus in terms of race, indigenous Africans have the lowest levels of access to basic services such as sanitation as they constitute the majority in informal settlements which are essentially spaces of exclusion and marginalization [4,5]. As this paper shows, levels of access in informal settlements are far below the national levels of access. Thus, the upgrading of informal settlements programme (UISP) has the provision of water, sanitation, and electricity to ensure the health and safety of the informal dwellers among its objectives [6]. These targets were articulated in the DHS strategy known as Outcome 8 [3]. The provision of basic services in informal settlements was articulated in the National Development Plan (Vision 2030) [7]. Thus, at a policy level, the provision of basic services is a key development priority. The range grants (Municipal Infrastructure grant) for improving access shows the importance that the government gives to reaching the masses with these services [3].

South Africa has three spheres of government, National, Provincial and Local government. Municipalities fall within the local government, which is the lowest level of the government spheres. Municipalities are an administrative division that may represent large urbanized regions which may encompass multiple cities or they may represent primarily rural areas. Since 1994 various policies and laws have been developed to guide the functions of municipalities. The 1996 Constitution, and through the Local Government Municipal Systems Act, Act No. 32 of 2000, states that municipalities are tasked with the duty to provide basic services such as water, sanitation, electricity and waste management [2,8]. In local government, the introduction of indigent tariffs sought to provide basic services to poor households that were excluded and could not afford to pay for basic services [9]. Since the end of apartheid significant progress has been made in service delivery [10]. According to the Community Survey of 2016 approximately 90% of households had access to piped water compared to 84% in 2001, access to a flush toilet increased from 49% in 2001 to 63% in 2016, the use of electricity increased from 57% in 1996 to 88% in 2016 and access to refuse removal services increased from 51% in 1996 to 64% in 2016 [11,12]. These figures point to service delivery improvements within South Africa.

Despite this progress, basic service delivery protests have continued to rise in post-apartheid South Africa [10,13]. Municipal IQ tracks the number of on-going protests against municipalities in South Africa and their data show that between 2004 and 2016 there have been 1225 service delivery protests [14]. Dissatisfaction with service delivery has often been cited as the reason for the increase in service delivery protests within South Africa [15,16]. Such dissatisfaction may be due to a lack of or poor service delivery or poor opinion over municipal management. The challenges faced by municipalities in the provision of basic services are perhaps due to the proliferation of informal settlements, also commonly referred to as slums in international literature [17]. Demographic, institutional and economic challenges vary across municipalities and provinces and affect the ability of municipalities to provide basic services [18]. It is therefore imperative to track the levels of access to basic services as well the levels of satisfaction with basic services in these settlements.

Citizen feedback is an important tool for assessing the adequacy, quality and efficiency of basic services delivery [19]. Such engagement with citizens facilitates the design and delivery of services based on the needs and priorities of citizens [20]. The nationally representative South African Social Attitudes Survey (SASAS) has been an important tool for tracking and evaluating government performance in the provision of various public services including but not limited to electricity, water, and sanitation and refuse removal. The informality of informal settlements means they are characterized by poor service delivery and profound inequalities in access to basic services. It is therefore not surprising that the 2015 SASAS report showed that informal settlement dwellers were the least satisfied with basic services delivery [21]. Dissatisfaction with basic services delivery can result in payment boycotts and negatively impacts social and economic development [9]. This suggests that an analysis of basic services delivery in informal settlements is a relevant research topic in South Africa.

Previous studies that assessed satisfaction with basic services in South Africa have not used nationally representative data but rather focused on one region [22,23,24]. A study by Masiya and team explored citizen’s satisfaction with basic municipal service delivery using the nationally representative 2016 South African Social Attitudes Survey (SASAS) [25]. However, this study did not specifically focus on informal settlements which bear the brunt of service delivery challenges. To the best of our knowledge, there has not been a nationally representative study that focuses on access to basic services and satisfaction with basic services with a focus on informal settlements in South Africa. In the present study, we aim to use data from a baseline assessment of South African Informal Settlements and multinomial logistic regression analysis to establish the determinants of satisfaction with basic services. This study sought to address this knowledge gap and not only provide the current status but also levels of satisfaction with basic services in informal settlements in South Africa. In South Africa, such a study is important, given the rising service delivery protests within informal settlements. An evaluation of satisfaction is an indicator of overall government performance and may be useful in influencing policy makers to identify service delivery challenges and gaps in delivery 

## 2. Materials and Methods

### 2.1. Data

**Figure 1:**
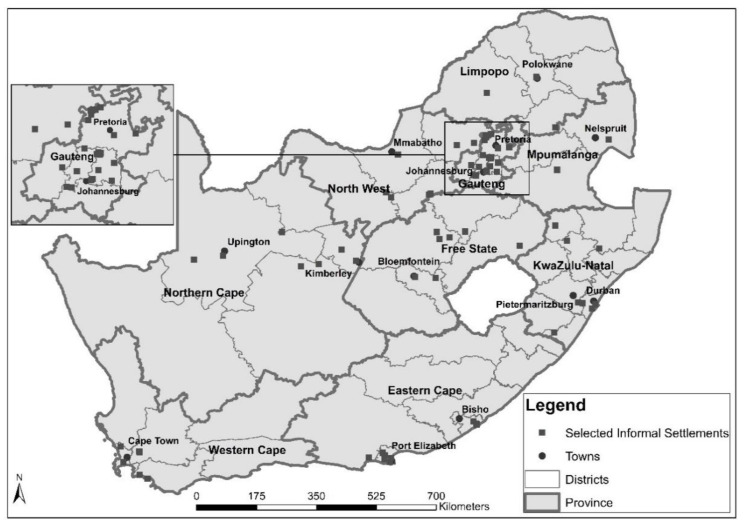
Selected/visited informal settlements in South Africa.

Data from a baseline assessment of South African Informal Settlements were used in this analysis. The study received ethics approval from the Research Ethics Committee (REC) of the HSRC (Research Ethics Committee Reference No: No REC 9/21/05/14). The survey was commissioned by the South African Department of Human Settlement in collaboration with the Department of Planning, Monitoring and Evaluation (DPME) during 2014–2015. The survey was conducted in informal settlements that were targeted for upgrading by the upgrading informal settlements programme (UISP). The study sought to create a baseline assessment database that would be used to track the development of communities in the UISP and conduct future impact evaluations. The survey applied a stratified random sampling approach to obtain a sample of nationally representative informal settlements targeted for upgrading by municipalities and metros. A total of 119 informal settlements were randomly selected from the 1185 settlements that were targeted for upgrading. With a fixed number of 45 households randomly selected from each informal settlement, a sample size of 5355 households was targeted. However, only 75 settlements and 2380 households were realized across the country due to time and budgetary constraints, as well as service delivery protests during data collection from June to September 2015. Although a 2% margin of error was used in the sample size calculation, the results are reliable at 3% margin of error. A detailed report of the survey methodology is provided elsewhere [26]. Figure 1 shows the distribution of the sampled informal settlements across the country.

### 2.2. Measures

The determinants of consumer satisfaction have been analyzed across various aspects such as electricity, health services provision, transport and sanitation [27,28,29,30,31]. In the current survey, the household questionnaire collected information on satisfaction with a range of community services and opportunities such as basic public services of water supply, sanitation, refuse and waste removal, transport links, housing, and employment and police services. This analysis focuses on satisfaction in basic service delivery of four key services-water, sanitation, electricity and refuse removal. Respondents were asked how satisfied they are with household water quality, sanitation services, frequency of electricity supply and waste management. A five-point Likert scale was used ranging from 1 being very dissatisfied to 5 being very satisfied. For the purposes of this analysis, our satisfaction variables were included as categorical variables 0—dissatisfied, 1—neutral and 2—satisfied. Respondents represent a head of household or acting head of household aged 18 years and older.

The selection of independent variables in our analysis was guided by existing literature [19,31,32]. Covariates in our analysis are consumer socio-demographic characteristics of the household head, household characteristics, community factors and service-related factors [19]. Based on the data available in our dataset, the characteristics of the household head included in our analysis were gender, age, race and education. Gender was included as a binary variable; 0—male and 1—female. None of the households’ heads reported being younger than 20 years of age. Age was categorized as 0—20 to 35 years, 1—36 to 60 years, and 3—61 years and older. Ethnicity data were collected in the categories of African, Colored, Asian/Indian, White and Other. Race was included as a binary variable with 0—African and 1—Non-African. Education was included as a categorical variable; 0—no education, 1—primary, 2—secondary, and 3—tertiary.

The household characteristics included in our study are wealth index and household participation in service delivery protests. The wealth index was constructed by applying Multiple Correspondence Analysis (MCA) to the household survey data. The household and living conditions considered in the analysis were housing type, electricity, refuse, water and sanitation services, and a set of thirteen household assets. The 13 household assets included are: fridge, DVD, vacuum cleaner, cell phone, washing machine, computer, internet access, electric or gas stove, television, telephone, radio, DSTV and motor vehicle. Respondents were also asked if they or any of the household members participated in a service delivery protest. Service delivery protest was entered as a binary variable; 0—no and 1—yes.

Our study also included a range of community factor variables. Respondents were asked to rate how safe they felt against criminals within the settlement. This variable was included as a categorical variable; 0—not safe, 1—fairly safe, 2—safe. Respondents were also asked to describe the quality of the relationship between the community and local government. This was included as a categorical variable; 1—good, 2—neither good nor bad and 3—bad. Respondents’ feeling regarding responsiveness of the municipality to the community’s needs was included as a binary variable; 1—not responsive, 2—responsive.

Our study also included various service-related factors for water, sanitation, refuse and electricity. Satisfaction with basic services is a function of consumer experiences and expectation regarding basic services delivery. For the basic service of water provision, we included factors such as the water source, distance from water source, whether the water was safe for drinking without any further treatment and if the household had to treat the water before drinking. Water source was included as categorical variable taking on the values of 1—piped tap water, 2—public communal tap, 3—neighbor, 4—water carrier, 5—other (which included borehole, flowing water, stagnant water, well and spring). For sanitation provision we considered factors such as the type of toilet facility, whether or not the toilet was shared and the location of the toilet. Type of toilet facility was included as a categorical variable; 1—none, 2—flush toilet, 3—chemical toilet, 4—pit latrine, 5—bucket and 5—other (which included nearby veld and any other). For refuse related services we included factors such as refusal disposal methods and whether refuse lying around was a problem in the area. Refuse disposal methods were included as categorical variables; 1—removed by local authority, 2—removed by community members contracted by municipality, 3—community members, 4—communal or own refuse dump, 5—dump anywhere, 6—burn it, 7—bury it, and 8—other. For electricity service-related factors, we included factors such as access to electricity and adequacy of electricity for lighting, cooking and heating. Access to electricity was included as a binary variable; 0—no and 1—yes.

### 2.3. Data Analysis

Statistical analysis was performed in STATA software version 13 [33]. Data were weighted in order to present results that are representative of the South African population living in informal settlements targeted for upgrading. Ordinary logistic models are not appropriate for the analysis of ordered outcomes. Previous literature has also shown the ordered logit models to be the most appropriate models in analysis that involves an ordered dependent variable when compared with the ordinary least square method [34]. As a result, ordered regression analysis has been commonly applied for analysis of ordinal variables [19]. One of the assumptions underlying the ordered logistic regression method is that the coefficients which describe the relationship between each pair of outcomes are the same. This is commonly referred to as the proportional odds assumption or the parallel regression assumption. The omodel command in STATA was used to test the proportional odds assumption. Our results show that our model violates the proportional odds assumption. Our study therefore makes use of a multinomial logistic regression analysis which has been used by previous studies that assessed satisfaction with various services [27,28]. The multinomial logistic regression is a useful model when the dependent variable is categorical with two or more outcomes or levels. The model assumes independence among these dependent variable categories and one of these levels is chosen as the reference category.

## 3. Results

### 3.1. Access to Basic Services Delivery in Informal Settlements—An Overview

**Figure 2:**
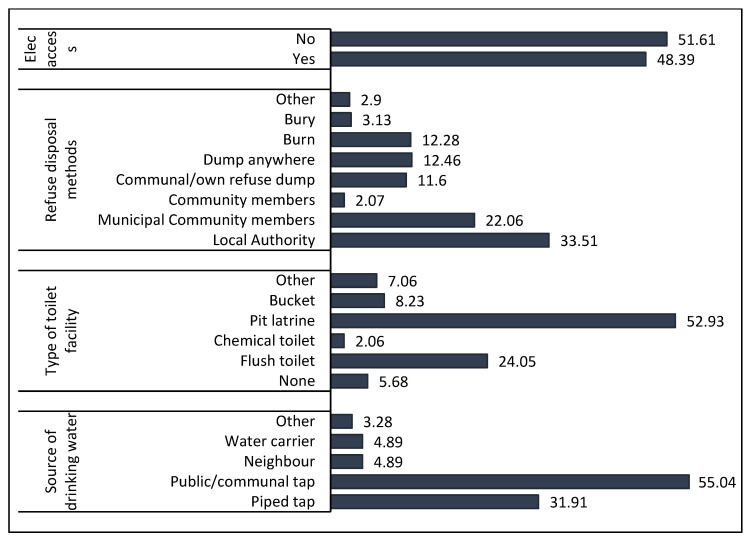
Access to basic services in informal settlements in South Africa.

Figure 2 shows access to basic services in the Informal Settlements Survey. The most common source of drinking water in the informal settlements is a public or communal tap (55%). Approximately 32% reported making use of a piped water tap to dwelling or site (yard). Less than 4% of respondents reported making use of other water sources such as a flowing water, stream, well, and spring water. The most common toilet facility was the pit latrine (53%) followed by a flush toilet (24%). Approximately 6% reported not having access to any toilet facility. More of the respondents reported that their refuse was removed by the local authorities (34%). The majority of the respondents in the study reported not having access to electricity (52%).

### 3.2. Descriptive Statistics

Table 1 shows survey weighted descriptive statistics for the study sample. Based on the individual characteristics our sample is predominantly African (96%), male (55%) and had secondary education (57%). A majority of the respondents reported that the communities they lived in were not safe (51%), and that their municipalities were rarely responsive (60%).

With regards to service-related characteristics a majority of the respondents were within 500 m to a water supply (74%), 94% thought water from their main source of water was safe for drinking and 93% reported they did not need to further treat water before drinking (see Table 2). Half of the respondents reported sharing a toilet facility with other households, and 45% of households reported that the toilets were not inside the dwelling but on the site (yard). More of the respondents thought refuse was a serious problem in the area (47%) than those who though it was a problem but not serious (24%) or thought it was not a problem (29%). Most of the respondents thought that electricity supply was not adequate for lighting (52%), cooking (51%) or heating (52%).

### 3.3. Satisfaction with Basic Services Provision

Table 3 provides an illustration of consumer satisfaction by wealth quintile. It is clear that satisfaction with basic services varies across the wealth quintiles. Whilst the majority of the respondents were satisfied with water quality (59%) a majority of respondents were dissatisfied with sanitation services (68%) refuse removal (55%) and frequency of electricity supply (62%). The prevalence of dissatisfaction with all services (water quality, sanitation services, refuse removal and electricity supply) was highest in the first quintile and lowest in the fifth quintile. The majority of individuals in the fifth quintile are dissatisfied with sanitation services and refuse removal. With regards to water quality and electricity supply a majority of the individuals from the fifth quintile are satisfied.

### 3.4. Determinants of Satisfaction with Service Delivery

Table 4 and Table 5 show results from the multinomial logistic regression analysis, using those who were dissatisfied as the reference category. Table 4 shows the results from the adjusted association of each variable with being neutral and Table 5 shows the results of the adjusted association of each variable with being satisfied.

#### 3.4.1. Water

In the multinomial regression for water quality, variables significantly associated with being neutral are age (Odds ratio [OR] 0.96; 95% Confidence Interval [CI] 0.94–0.99), being female versus being male (0.58; 0.36–0.94), secondary education (0.42; 0.17–1.02) and tertiary education versus no education (0.26; 0.07–1.06). Middle wealth index quintile (4.14; 1.89–9.09), higher wealth index quintile (2.40; 0.99–5.80) and highest wealth index quintile (3.95; 1.62–9.59) versus lowest wealth index quintile were also found to be significant. Other demographic variable that are significantly associated with being neutral are a bad relationship with government versus good relationship with government (0.46; 0.24–0.91), living in a fairly safe community (0.52; 0.27–0.99) and living in a safe community (0.19; 0.08–0.42) versus an unsafe community.

The service-related factors results show that individuals using a neighbor’s tap (0.18; 0.03–1.23) are less likely to be neutral regarding water quality than those using piped water source. Those who reported water as being safe for drinking were more likely than those who reported water being unsafe for drinking to report neutral satisfaction rather than being dissatisfied by water quality (5.65; 1.91–16.67). Individuals who report that they never have to further treat water are less likely than those who report always having to further treat water to be neutral regarding water quality (0.41; 0.16–1.08).

Variables significantly associated with being satisfied with water quality were age (0.98; 0.97–0.99), tertiary education (0.42; 0.18–0.95) versus no education. Lower wealth index quintile (1.58; 1.10–2.28), middle wealth index quintile (1.56; 1.07–2.27), higher wealth index quintile (1.89; 1.27–2.79), and highest wealth index quintile (2.49; 1.64–3.78) versus lowest wealth index quintile were also found to be significant. Other variables that are significantly associated with being neutral are participating in a service delivery protest versus not participating (0.78; 0.60–1.02), living in a fairly safe community versus an unsafe community (1.90; 1.40–2.59), living in a safe community versus an unsafe community (1.36; 1.01–1.82), neutral (0.41; 0.30–0.55) and bad (0.33; 0.23–0.46) relationship with government versus good relationship with government.

Individuals using a piped water source were more likely to be satisfied than individuals using a public tap (0.25; 0.17–0.37) or a neighbor’s tap (0.22; 0.12–0.40) or water carrier (0.50; 0.26–0.95) or any other water source (0.29; 0.15–0.57) Individuals who report their water source is outside the dwelling and a distance of less than 500 m to a water source are more likely than individuals who report having water source inside their dwelling to be satisfied with water quality (1.38; 0.95–2.00). Those that report a distance of more than one kilometer are less likely than those who have the water source inside their dwelling to report being satisfied than dissatisfied (0.10; 0.02–0.49). Individuals who report water as being safe for drinking are more likely than those who report water as being unsafe to report being satisfied with water quality (18.08; 9.11–35.89). Individuals who report that they never have to further treat water are more likely than those who report always having to further treat water to be satisfied with water quality (4.52; 2.19–9.30).

#### 3.4.2. Sanitation Provision

In the multinomial regression for sanitation services, variables significantly associated with being neutral are age (0.98; 0.96–1), being non-African versus African (0.41; 0.14–1.19), tertiary education versus no tertiary education (0.28; 0.07–1.05), higher wealth index versus lowest wealth index quintile (1.71; 0.94–3.11). From the service-related factors, the results show that those who use a flush toilet (0.20; 0.06–0.60), or chemical toilet (0.19; 0.05–0.80), or pit latrine (0.04; 0.01–0.12), or bucket (0.07; 0.02–0.22), or other (0.08; 0.02–0.32) were less likely than those not making use of any toilet facility to report being neutral. Those who reported that their toilet was located on site (in yard) (5.05; 2.84–8.96) were more likely than those who reported their toilet facility was inside the dwelling to report being neutral.

Variables significantly associated with being satisfied were female (0.47; 0.35–0.64), non-African (0.52; 0.25–1.06), primary (0.46; 0.26–0.82), secondary (0.33; 0.18–0.61) and tertiary (0.18; 0.07–0.48) education versus no education, and individuals in the lower (2.19; 1.18–4.05), higher (2.02; 1.10–3.72), or highest (2.35; 1.26–4.39) wealth index quintile versus lowest wealth index quintile. Other variables significantly associated with being satisfied were individuals who reported protesting to service delivery (1.70; 1.21–2.38), individuals who reported their community was fairly safe (1.46; 1.02–2.11) or safe (2.75; 1.91–3.95), and individuals who reported having a neutral relationship with the government (0.72; 0.51–1.04), or a bad relationship with the government (0.40; 0.26–0.61). From the service-related factors, the results show that those who use a flush toilet (7.61; 1.14–50.58) were more likely than those not making use of any toilet facility to report being satisfied than dissatisfied. Those who reported sharing a toilet (0.38; 0.27–0.54) were less likely than those who reported not sharing a toilet to report being satisfied with sanitation services versus being dissatisfied. Surprisingly, those who reported that their toilet was located on site (in yard) (3.92; 2.60–5.93) were more likely than those who reported their toilet facility was inside the dwelling to report being satisfied. This might be due to the poor quality of the toilets that were inside the dwellings.

#### 3.4.3. Refuse Removal

In the multinomial regression for refuse removal, the variables significantly associated with being neutral are female (0.56; 0.41–0.76) versus being male, being non-African versus being African (0.44; 0.18–1.05), primary education (2.29; 1.12–4.69), secondary education (3.09; 1.47–6.46) versus non education, lower wealth index versus lowest wealth index quintile (0.57; 0.35–0.93), having participated in a service delivery protest versus not participating in one (0.73; 0.51–1.04), and a bad relationship with the government versus a good relationship (0.36; 0.23–0.56). Service-related factors associated with being neutral were community members contracted by the municipality to remove refuse (3.20; 2.16–4.7), dumping refuse anywhere (0.15; 0.07–0.34), burning refuse (0.57; 0.34–97) and burying refuse (0.04; 0.00–0.35) versus refuse removal by the municipality; reporting refuse was not a problem (4.58; 3.09–6.78), versus reporting refuse is a serious problem.

Variables significantly associated with being satisfied were female (0.67; 0.50–0.89), tertiary education versus no education (0.36; 0.13–1.02), individuals in the lower (1.64; 0.99–2.72), middle (1.92; 1.16–3.19), higher (3.44; 2.09–5.66) or highest (4.31; 2.55–7.29) wealth index quintile versus lowest wealth index quintile, and individuals who reported neutral government relationship (0.73; 0.51–1.04) or a bad relationship with the government (0.42; 0.28–0.63) versus a good relationship. Service-related factors associated with being satisfied were using a communal or own refuse dump (0.26; 0.16–0.42), dumping refuse anywhere (0.04; 0.01–0.11), burning refuse (0.07; 0.03–0.15) and burying refuse (0.13; 0.06–0.33) versus refuse removal by the municipality.

#### 3.4.4. Electricity Provision

In the multinomial regression for frequency of electricity supply, reporting that the municipality is responsive to the community’s needs versus reporting that the municipality is not responsive to the community’s needs (1.93; 1.18–3.16) was significantly associated with being neutral. Service-related factors associated with being neutral were access to electricity (2.75; 1.40–5.40) versus no electricity access.

Variables significantly associated with being satisfied were living in being non-African versus being African (1.90; 1.25–2.89), middle (4.86; 1.55–15.26), higher (4.22; 1.31–13.60) or highest (9.81; 3.03–31.81)) wealth index quintile versus lowest wealth index, reporting that the community is safe (1.59; 1.10–2.30) versus reporting that the community is not safe, and reporting a neutral (0.56; 0.38–0.83) or bad relationship with the government (0.48; 0.32–0.72) versus a good relationship. Service-related factors associated with being satisfied were access to electricity (13.34; 7.30–24.38) versus no electricity access, inadequate electricity access for lighting versus adequate electricity access for lighting (0.46; 0.23–0.90), inadequate electricity access for cooking versus adequate electricity access for cooking (0.44; 0.21–0.89), and inadequate electricity access for heating versus adequate electricity access for heating (1.81; 0.98–3.34).

## 4. Discussion

In this paper, we examined the status of basic services delivery and satisfaction with basic services delivery in informal settlements targeted for upgrading. The importance of a study of this nature is reflected in the SDGs targeted for 2030. Access to basic services is a key component across many of the SDGs. Amongst these, SDG 1 target 4 seeks to ensure that the poor and vulnerable have access to basic services. SDG 6 seeks to ensure the availability of water and sanitation for all. SDG 7 seeks to ensure access to affordable and sustainable modern energy. SDG 11 target 1 seeks to ensure access to adequate, safe and affordable basic services and the upgrade of slums. Our results provide important indicators for these targets.

Whilst the South African government has made noteworthy progress in addressing the many challenges faced by those who reside in informal settlements, our results are consistent with others in showing inadequate access to basic services in these areas. Consistent with previous studies, our results showed that a communal or public tap was the most common water source within the informal settlements, over 50% of the respondents made use of pit latrines, and over 50% did not have access to electricity [35]. Progress in the delivery of basic services in informal settlements is normally outrun by rural to urban migration; therefore, the demand for basic services outstrips supply [10]. According to 2017 World Bank data, close to two thirds of South Africa’s population lived in urban areas, compared to 50% in 1994 [36]. These population increases place pressure on basic service delivery within urban areas and attainment of the SDGs. Nengwekhulu reported that the challenges South Africa faces in the delivery of basic services are not only a result of the continued mushrooming of informal settlements, but also a result of various factors such as shortage of skills, bureaucratic work ethic in the public sector, growing political interference and nepotism [10].

Our study also sought to determine the levels of satisfaction with basic services delivery in the informal settlements. Previous research has shown that satisfaction with basic services delivery in South Africa is generally low [32]. Using the SASAS data, Bohler-Muller and team found that dissatisfaction levels with basic service delivery are generally higher in informal settlements when compared to urban formal and rural areas [21]. Informal settlement dwellers were particularly dissatisfied with electricity provision. Approximately 62% of informal settlement dwellers reported being dissatisfied with electricity provision [21]. Consistent with these findings, our study also reported that 62% of respondents were dissatisfied with electricity provision. In our study, respondents held more negative feelings about sanitation with over 68% reporting dissatisfaction compared to 58% who reported dissatisfaction with water and sanitation in the 2015 SASAS survey [21]. Approximately 31% of respondents in our study were satisfied with refuse removal. This number is lower than findings from a study by Masiya and team who found a higher number of respondents satisfied with refuse removal (53%) [25]. These differences are probably attributable to differences in the data used, our study focused on informal settlements data only, whilst the study by Masiya et al. [25] used data from South Africa as a whole.

We also assessed the factors within informal settlements that contribute to satisfaction with basic services delivery. From the demographic and community factors included in the multinomial regression we found that the wealth index and the quality of the relationship between the community and government were important factors for satisfaction with basic services. Our study showed that those who had a bad relationship with government were less likely to report being satisfied with basic services. This is consistent with findings from a Cape Town based study which also found that individuals who reported poor government engagement with the Khayelitsha community also reported dissatisfaction with basic service delivery [37]. Our study also showed that individuals within the highest wealth index were more likely than individuals in the lowest to report being satisfied than dissatisfied with basic services. This finding is consistent with a study that also finds that the non-poor are more satisfied with basic services when compared to poor households [25].

Our study also shows various service-related factors that are associated with basic services delivery. In our study, satisfaction with water was associated with the service-related factors of type of water source, distance to water source, safety of water for drinking and further treatment of water by households. For example, respondents who reported that water was safe for drinking were more likely than respondents who reported water was not safe for drinking to be satisfied with water quality. These findings are intuitive and consistent with previous studies that show that service-related factors such as water colour or water foul smell are associated with satisfaction in water service delivery [38]. Service-related factors of toilet location; toilet facility type and toilet sharing were significantly associated with satisfaction in sanitation services. For example, individuals who shared a toilet were less likely than individuals who did not share a toilet to report being satisfied with sanitation services than dissatisfied. A study by Islam and Khan finds that service-related factors such as the cleanliness of common toilets is associated with dissatisfaction [38]. Refuse disposal methods and refuse problems within communities were significant predictors of satisfaction with refuse removal services. For example, individuals who reported refuse was not a problem within the community were more likely than those who reported it was not a problem to report being satisfied with refuse removal than dissatisfied. Consistent with findings by Islam and Khan, the collection of garbage by municipalities resulted in greater satisfaction [38]. Satisfaction with electricity supply was significantly associated with access to electricity and adequacy of electricity for cooking and heating. Results show that individuals who reported having access to electricity were more likely than those without electricity supply to report being satisfied with electricity provision than dissatisfied.

Consumer dissatisfaction often leads to the unwillingness to pay for basic services. Although governmental transfers are one source of municipal funding, municipalities are required to be mostly self-financing and raise a reasonable amount of their revenue from services charges, rates and taxes [39]. The non-payment of basic services hampers municipal cost recovery and municipal financial viability [9,40]. Therefore, amongst other factors such as improving municipal administration and billing systems, consumer dissatisfaction is a possible driver of non-payment, and, therefore, municipal revenue depletion [9].

Our study has some limitations. Firstly, the use of cross-sectional dataset limits any causal interpretations. Drawing on other datasets may help in establishing how people’s opinions have changed over time. As noted in the methods section, due to time and budgetary constraints not all sampled settlements were visited and only informal settlements that were targeted for upgrading were visited. This may mean our results may not be an accurate representation of basic service delivery and satisfaction with basic service delivery across the country’s informal settlements. Future research should consider these shortcomings.

## 5. Conclusions

Our results are an important contribution to the literature on basic services in South African informal settlements. This study provided an overview of basic service delivery in informal settlements and assessed the factors associated with satisfaction to basic service delivery. Our study shows that those living in these informal settlements experience inadequate access to basic services. Satisfaction with basic services varied by socio-economic status. Respondents held low levels of satisfaction with sanitation provision, refuse removal and electricity provision. In general terms, the proportion of respondents who were satisfied with the public service delivery of water quality was high. Various factors were also identified that are important in ensuring that services delivery meets the expectations of citizens. The study highlights the need to reduce the distance to water sources, address refuse problems, improve access to non-shared toilet facility per household and electricity. Understanding the levels of basic services delivery satisfaction is an important tool for assessing the adequacy and efficiency of basic services delivery. Consumer satisfaction is an important driver of services delivery payment by consumers, which directly influences the ability of municipalities to provide basic services.

## Figures and Tables

**Table 1:** Demographic characteristics of study sample.

Variable	Number	%
Individual characteristics		
Age (mean +/− s.d.)	45.11	12.94
Gender		
Male	1222	54.66
Female	1014	45.34
Race		
African	2241	96.31
Non-African	85	3.69
Education		
None	224	10.16
Primary	654	29.58
Secondary	1254	56.69
Tertiary	79	3.57
Household characteristics		
Wealth index		
Lowest	505	21.25
Lower	446	18.75
Middle	475	20.00
Higher	476	20.03
Highest	474	19.96
Protest		
No	1677	74
Yes	589	26
Community factors		
Safety		
Not safe	1198	50.97
Fairly safe	573	24.38
Safe	579	24.64
Government relationship		
Good	816	34.87
Neutral	743	31.75
Bad	782	33.38
Municipal responsive		
Not responsive	1398	59.74
Responsive	941	40.26

Note: All estimates are weighted, s.d.-Standard deviation.

**Table 2:** Service-related factors.

Variable Description	Number	%
Distance to water supply		
1 = In-dwelling	515	22.28
2 ≤ 500 m	1719	74.31
3 = 501–1000 m	40	1.77
4 ≥ 1 km	38	1.65
Water safe for drinking		
0 = No	148	6.42
1 = Yes	2171	93.58
Household further treats water		
1 = Yes always	93	4.02
2 = Yes sometimes	63	2.72
3 = No, never	2160	93.26
Toilet shared		
0 = No	1074	49.66
1 = Yes	1088	50.34
Toilet location		
1 = In dwelling	469	21.76
2 = On site (in yard)	976	45.29
3 = Off site	710	32.95
Refuse problem		
1 = A serious problem	1057	47.03
2 = A problem but not serious	544	24.21
3 = Not a problem	646	28.76
Electricity adequate for:		
Lighting		
1 = Adequate	1111	48.2
2 = Not adequate	1194	51.8
Cooking		
1 = Adequate	1107	48.51
2 = Not adequate	1174	51.49
Heating		
1 = Adequate	1072	47.62
2 = Not adequate	1179	52.38

Note: All estimates are weighted.

**Table 3:** Satisfaction with basic services by wealth quintile (percent).

Basic Service	Income Quintile					
Water Quality	1	2	3	4	5	Total
Dissatisfied	50.62	37.06	34.96	31.14	22.55	35.53
Neutral	3.25	3.93	8.56	5.05	4.78	5.11
Satisfied	46.13	59	56.47	63.81	72.67	59.35
Sanitation						
Dissatisfied	86.85	69.67	67.29	60.42	55.69	68.24
Neutral	6.3	9.48	11.98	11.97	7.75	9.48
Satisfied	6.85	20.85	20.73	27.61	36.55	22.28
Refuse Removal						
Dissatisfied	72.65	60.41	51.84	44.92	43.94	55
Neutral	13.41	13.88	15.51	12.73	16.65	14.41
Satisfied	13.94	25.71	32.66	42.35	39.41	30.58
Electricity						
Dissatisfied	97.33	93.89	60.23	41.74	23.56	62.21
Neutral	2.06	3.28	9.77	12.32	6.52	6.9
Satisfied	0.61	2.83	30	45.94	69.92	30.89

Note: All estimates are weighted.

**Table 4:** Logistic regression analysis of factors associated with neutral satisfaction in basic service.

Variable	Water			Sanitation			Refuse			Electricity		
	OR	Conf. Int.		OR	Conf. Int.		OR	Conf. Int.		OR	Conf. Int.	
Individual Characteristics												
Age (mean +/− s.d.)	0.9631 ***	0.9409	0.9858	0.9820 **	0.9664	0.9979	1.0006	0.9867	1.0147	0.9992	0.9810	1.0177
Gender												
Male												
Female	0.5806 **	0.3605	0.9350	0.7553	0.5315	1.0733	0.5576 ***	0.4084	0.7612	1.0880	0.7107	1.6657
Race												
African												
Non-African	1.2615	0.4049	3.9300	0.4067 *	0.1393	1.1874	0.4385 *	0.1823	1.0548	1.0605	0.5212	2.1582
Education												
None												
Primary	0.5273	0.2205	1.2608	0.6664	0.3124	1.4213	2.2874 **	1.1155	4.6904	0.9314	0.4243	2.0444
Secondary	0.4160 **	0.1692	1.0226	0.6521	0.3017	1.4097	3.0860 ***	1.4744	6.4595	0.9623	0.4226	2.1914
Tertiary	0.2648 *	0.0659	1.0633	0.2792 **	0.0742	1.0504	1.1687	0.3694	3.6973	1.3478	0.3073	5.9115
Household characteristics												
Wealth index												
Lowest												
Lower	1.6343	0.6766	3.9475	1.4851	0.8119	2.7165	0.5723 **	0.3506	0.9341	1.0222	0.4440	2.3533
Middle	4.1418 ***	1.8871	9.0901	1.0477	0.5703	1.9246	0.7315	0.4495	1.1906	1.8953	0.8462	4.2451
Higher	2.3972 **	0.9907	5.8004	1.7111 *	0.9426	3.1061	0.9467	0.5804	1.5442	1.5913	0.6392	3.9613
Highest	3.9450 ***	1.6225	9.5918	1.1867	0.6135	2.2954	1.2806	0.7669	2.1382	1.9916	0.7506	5.2843
Protest												
No												
Yes	1.1127	0.6621	1.8701	1.0372	0.6986	1.5398	0.7262 *	0.5085	1.0371	0.8300	0.5025	1.3710
Community factors												
Safety												
Not safe												
Fairly safe	0.5217 **	0.2747	0.9906	1.0227	0.6698	1.5615	1.0124	0.6933	1.4783	1.1445	0.6826	1.9192
Safe	0.1851 ***	0.0820	0.4177	1.4266	0.9190	2.2146	0.8983	0.6020	1.3405	1.4344	0.8463	2.4314
Government relationship												
Good												
Neutral	0.7824	0.4308	1.4208	1.4341	0.9282	2.2158	0.9045	0.6186	1.3224	1.1366	0.6632	1.9477
Bad	0.4634 **	0.2362	0.9092	1.0128	0.6167	1.6635	0.3600 ***	0.2295	0.5646	0.7627	0.4139	1.4055
Municipal responsive												
Not responsive												
Responsive	1.4722	0.8622	2.5137	1.2168	0.8099	1.8284	1.1196	0.7798	1.6073	1.9291 ***	1.1774	3.1608
Service-related factors												
Water source												
Piped tap												
Public/communal tap	0.8031	0.3902	1.6528									
Neighbor	0.1773 *	0.0256	1.2299									
Water carrier	1.4291	0.4024	5.0754									
Other	0.1672	0.0164	1.7016									
Distance to water supply												
1 = In-dwelling												
2 = < 500 m	0.8855	0.4128	1.8998									
3 = 501–1000 m	0.0539	0.0001	26.7990									
4 = > 1 km	0.1097	0.0051	2.3760									
Water safe for drinking												
0 = No												
1 = Yes	5.6480 ***	1.9135	16.6710									
Household further treats water												
1 = Yes always												
2 = Yes sometimes	1.0487	0.2851	3.8569									
3 = No, never	0.4129 *	0.1575	1.0827									
Type of toilet facility												
None												
Flush toilet				0.1964 ***	0.0643	0.5998						
Chemical toilet				0.1932 **	0.0473	0.7900						
Pit latrine				0.0393 ***	0.0128	0.1206						
Bucket				0.0680 ***	0.0208	0.2227						
Other				0.0824 ***	0.0216	0.3152						
Toilet shared												
0 = No												
1 = Yes				0.9208	0.6111	1.3874						
Toilet location												
1 = In dwelling												
2 = On site (in yard)				5.0503 ***	2.8477	8.9567						
3 = Off site				1.6296	0.8888	2.9876						
Refuse disposal methods												
Local Authority												
Municipal Community members							3.1976 ***	2.1612	4.7311			
Community members							0.8133	0.2529	2.6151			
Communal/own refuse dump							0.7890	0.4762	1.3074			
Dump anywhere							0.1547 ***	0.0698	0.3427			
Burn							0.5717 **	0.3360	0.9730			
Bury							0.0382 ***	0.0042	0.3496			
Other							0.6983	0.2190	2.2269			
Refuse problem												
1 = A serious problem												
2 = A problem but not serious							1.3879	0.9291	2.0733			
3 = Not a problem							4.5803 ***	3.0939	6.7810			
Electricity access												
No												
Yes										2.7523 ***	1.4035	5.3973
Electricity adequate for:												
Lighting												
1 = Adequate												
2 = Not adequate										0.5352	0.2133	1.3430
Cooking												
1 = Adequate												
2 = Not adequate										1.8353	0.6834	4.9289
Heating												
1 = Adequate												
2 = Not adequate										0.9219	0.3697	2.2988

Notes: Dissatisfied is the reference category, *** *p* < 0.01; ** *p* < 0.05; * *p* < 0.1, Conf.Int.-95% Confidence Interval, OR-Odds Ratio.

**Table 5:** Logistic regression analysis of factors associated with satisfaction in basic services.

Variable	Water			Sanitation			Refuse			Electricity		
	OR	Conf. Int.		OR	Conf. Int.		OR	Conf. Int.		OR	Conf. Int.	
Individual characteristics												
Age (mean +/− s.d)	0.9820 ***	0.9713	0.9928	0.9973	0.9843	1.0105	1.0100	0.9974	1.0229	1.0029	0.9903	1.0158
Gender												
Male												
Female	0.8246	0.6501	1.0459	0.4733 ***	0.3505	0.6391	0.6697 **	0.5034	0.8908	1.0596	0.7895	1.4220
Race												
African												
Non-African	0.8258	0.4190	1.6273	0.5184 *	0.2527	1.0633	0.6575	0.3159	1.3683	1.8995 ***	1.2497	2.8873
Education												
None												
Primary	1.2342	0.7735	1.9693	0.4621 ***	0.2606	0.8196	0.8895	0.5187	1.5256	0.7388	0.4288	1.2730
Secondary	0.9988	0.6160	1.6195	0.3344 ***	0.1840	0.6077	1.4285	0.8169	2.4978	0.7558	0.4282	1.3339
Tertiary	0.4214 **	0.1863	0.9533	0.1847 ***	0.0705	0.4837	0.3632 **	0.1289	1.0229	1.1704	0.4182	3.2754
Household characteristics												
Wealth index												
Lowest												
Lower	1.5819 **	1.0994	2.2761	2.1896 **	1.1823	4.0548	1.6425 **	0.9903	2.7242	2.2418	0.6750	7.4454
Middle	1.5587 **	1.0717	2.2672	1.2768	0.6899	2.3632	1.9237 **	1.1609	3.1877	4.8563 ***	1.5459	15.2554
Higher	1.8862 ***	1.2737	2.7932	2.0213 **	1.0972	3.7235	3.4355 ***	2.0852	5.6603	4.2249 ***	1.3125	13.6002
Highest	2.4894 ***	1.6415	3.7752	2.3537 ***	1.2606	4.3949	4.3084 ***	2.5477	7.2860	9.8106 ***	3.0256	31.8115
Protest												
No												
Yes	0.7816 *	0.5971	1.0232	1.7018 ***	1.2163	2.3811	0.7807	0.5648	1.0791	0.7795	0.5536	1.0976
Community factors												
Safety												
Not safe												
Fairly safe	1.9016 ***	1.3973	2.5878	1.4675 **	1.0224	2.1061	1.3194	0.9405	1.8510	0.8922	0.6266	1.2705
Safe	1.3551 **	1.0117	1.8150	2.7482 ***	1.9100	3.9543	1.1489	0.7971	1.6560	1.5899 **	1.1002	2.2976
Government relationship												
Good												
Neutral	0.4064 ***	0.2988	0.5530	0.7249 **	0.5070	1.0363	0.7280 *	0.5106	1.0380	0.5581 ***	0.3754	0.8296
Bad	0.3252 ***	0.2318	0.4564	0.4020 ***	0.2630	0.6146	0.4239 ***	0.2840	0.6326	0.4752 ***	0.3153	0.7162
Municipal responsive												
Not responsive												
Responsive	0.9783	0.7414	1.2909	0.8882	0.6314	1.2494	1.0489	0.7528	1.4616	1.1090	0.7831	1.5704
Service-related factors												
Water source												
Piped tap												
Public/communal tap	0.2513 ***	0.1730	0.3652									
Neighbor	0.2174 ***	0.1181	0.4002									
Water carrier	0.5009 **	0.2641	0.9501									
Other	0.2915 ***	0.1500	0.5667									
Distance to water supply												
1 = In-dwelling												
2 = < 500 m	1.3805 *	0.9510	2.0039									
3 = 501–1000 m	0.7897	0.2801	2.2265									
4 = > 1 km	0.1076 ***	0.0237	0.4891									
Water safe for drinking												
0 = No												
1 = Yes	18.0821 ***	9.1108	35.8873									
Household further treats water												
1 = Yes always												
2 = Yes sometimes	1.4867	0.5184	4.2636									
3 = No, never	4.5182 ***	2.1941	9.3040									
Type of toilet facility												
None												
Flush toilet				7.6087 **	1.1444	50.5875						
Chemical toilet				1.3834	0.1640	11.6697						
Pit latrine				0.3788	0.0567	2.5307						
Bucket				0.2346	0.0321	1.7166						
Other				1.2868	0.1716	9.6473						
Toilet shared												
0 = No												
1 = Yes				0.3778 ***	0.2652	0.5383						
Toilet location												
1 = In dwelling												
2 = On site (in yard)				3.9265 ***	2.6019	5.9255						
3 = Off site				1.3439	0.8547	2.1131						
Refuse disposal methods												
Local Authority												
Municipal Community members							0.9841	0.6935	1.3964			
Community members							0.5408	0.2195	1.3324			
Communal/own refuse dump							0.2563 ***	0.1556	0.4224			
Dump anywhere							0.03804 ***	0.0129	0.1119			
Burn							0.0716 ***	0.0349	0.1469			
Bury							0.1349 ***	0.0553	0.3289			
Other							1.1637	0.5052	2.6808			
Refuse problem												
1 = A serious problem												
2 = A problem but not serious							1.8789 ***	1.3080	2.6991			
3 = Not a problem							6.9309 ***	4.8380	9.9292			
Electricity access												
No												
Yes										13.3396 ***	7.2982	24.3820
Electricity adequate for:												
Lighting												
1 = Adequate												
2 = Not adequate										0.4555 **	0.2315	0.8963
Cooking												
1 = Adequate												
2 = Not adequate										0.4359 **	0.2127	0.8933
Heating												
1 = Adequate												
2 = Not adequate										1.8131 **	0.9846	3.3388

Notes: Dissatisfied is the reference category, *** *p* < 0.01; ** *p* < 0.05; * *p* < 0.1, Conf.Int.-95% Confidence Interval, OR-Odds Ratio.
